# Long-term anakinra therapy in Schnitzler syndrome: real-world evidence on remission of neutrophilic dermatitis and systemic inflammation, safety, biomarker insights and dose optimization

**DOI:** 10.3389/fimmu.2026.1830866

**Published:** 2026-05-04

**Authors:** Mariusz Sikora, Marcin Ziętkiewicz, Karina Jahnz-Różyk, Ewa Więsik-Szewczyk

**Affiliations:** 1 Department of Internal Medicine, Pneumonology, Allergology, Clinical Immunology and Rare Diseases, Military Institute of Medicine - National Research Institute, Warsaw, Poland; 2 National Institute of Geriatrics, Rheumatology and Rehabilitation, Warsaw, Poland; 3 Department of Rheumatology, Clinical Immunology, Geriatrics and Internal Medicine, Medical University of Gdansk, Gdansk, Poland

**Keywords:** anakinra, autoinflammatory diseases, biomarkers, interleukin-1 blockade, neutrophilic dermatitis, serum amyloid A, Schnitzler syndrome

## Abstract

**Introduction:**

Schnitzler syndrome (SchS) is a rare, late-onset, acquired autoinflammatory disorder characterized by recurrent urticarial lesions, monoclonal gammopathy and systemic inflammation. Although interleukin-1 (IL-1) blockade with anakinra is considered as a standard of care, real-world data on long-term outcomes, biomarker utility and dose reduction remain limited. To evaluate the long-term efficacy and safety of anakinra, the utility of inflammatory biomarkers for disease monitoring, the feasibility of dose reduction and diagnostic delay in a cohort of Polish patients with SchS.

**Methods:**

We conducted a retrospective observational study of 13 adults with SchS treated with anakinra at two Polish tertiary referral centers between 2018 and 2025. Clinical manifestations, laboratory parameters, treatment response, adverse events and longitudinal follow-up data were analyzed. Receiver operating characteristic (ROC) analyses were used to compare biomarkers of disease activity, including serum amyloid A (SAA), C-reactive protein (CRP), erythrocyte sedimentation rate (ESR), fibrinogen, D-dimers and complete blood count (CBC)-derived inflammatory indices.

**Results:**

All 13 patients achieved rapid complete clinical remission within 48 hours of starting anakinra, accompanied by significant reductions in CRP and SAA. Remission was sustained over a median follow-up of 38.2 months (maximum 92.5 months), with no treatment discontinuations. In 3 patients (23%), dosing intervals were successfully extended to every 48–72 hours without loss of disease control. ROC analyses revealed that SAA showed the highest discriminatory power for distinguishing active disease from remission (AUC 0.974), outperforming ESR, fibrinogen and D-dimers. CBC-derived inflammatory indices declined markedly after treatment initiation but served mainly as adjunctive rather than primary monitoring tools. The therapy was well tolerated with no serious adverse events. The most frequent events were injection-site reactions, mild paradoxical psoriasis, transient neutropenia and consistent weight gain. Median diagnostic delay was 49 months.

**Conclusions:**

Anakinra provides highly effective and safe long-term disease control in SchS. SAA appears to be the most reliable biomarker for monitoring disease activity, while cautious interval prolongation may be feasible in selected patients in sustained remission. Furthermore, treatment-associated weight gain is a consistent clinical observation requiring attention.

## 1 Introduction

Schnitzler syndrome (SchS) is a rare, acquired, late-onset autoinflammatory disorder. It is characterized by the coexistence of a recurrent urticarial lesions and monoclonal gammopathy (predominantly IgM), often accompanied by systemic inflammatory manifestations including recurrent fever, bone pain, lymphadenopathy and elevated inflammatory markers (1). The condition was first described by the French dermatologist Liliane Schnitzler in 1972 (2). Epidemiologically, SchS typically manifests in middle-aged to older adults, with the age of onset most commonly in the sixth decade of life and exhibits a slight male predominance (1, 3). It is an extremely rare condition globally. Although more than 300 cases have been documented in the literature to date (3), its true prevalence is likely higher. There is a high likelihood of underdiagnosis and misdiagnosis due to the significant clinical overlap of SchS with more common dermatological, rheumatological and hematological conditions. Despite the disease having been recognized for over half a century with established and validated diagnostic criteria, its diagnosis remains challenging and often delayed (4, 5). All symptoms pose a significant impact on patients quality of life (6). Moreover, the monoclonal component carries a risk of progression to a hematologic malignancy, as well as rare cases of AA amyloidosis have also been reported (7–9).

The pathogenesis of SchS is primarily driven by dysregulated interleukin-1 signaling (10). Although IL-1 blockade produces prompt improvement, the exact mechanism underlying the interplay between the monoclonal protein and the aberrant inflammasome activation still remains not fully elucidated (1, 11).

Historically, before the development of IL-1 targeted therapies, treatment options for SchS were limited and largely ineffective. Conventional immunosuppressants (systemic glucocorticosteroids, colchicine, methotrexate), antihistamines and even biologics targeting other cytokines provided at most transient or partial response (12). The introduction of anakinra, a recombinant human IL-1 receptor antagonist (IL-1ra), provides rapid and sustained remission in the vast majority of patients. This substantial therapeutic advancement has shifted the paradigm of SchS management, establishing IL-1 blockade as the standard of care (13). Beyond anakinra, other IL-1 inhibitors including canakinumab (monoclonal antibody against IL-1β) and rilonacept (soluble decoy receptor for IL-1), have also demonstrated efficacy in SchS (14, 15).

Despite this therapeutic progress, significant knowledge gaps persist. While the evidence base has expanded beyond case reports and small case series to include larger cohorts and multinational registries, there is an ongoing need for real-world longitudinal studies to better understand treatment maintenance and monitoring in clinical practice. The aim of the present study is to address these unmet needs by presenting a real-world experience with anakinra in a cohort of SchS patients from reference centers. We specifically focus on:

1. The long-term efficacy of anakinra in controlling manifestations of SchS and its safety profile over prolonged exposure,
2. The comparative analysis of inflammatory biomarkers as tools for monitoring and detecting subclinical disease activity,
3. The feasibility and safety of dose reduction, including tapering and interval prolongation with their clinical implications,
4. The significant diagnostic odyssey faced by patients, often leading to delayed diagnosis.

## 2 Methods

### 2.1 Study design and patient population

We performed a retrospective, observational study of patients diagnosed with SchS, who were treated with anakinra at tertiary reference centers for autoinflammatory disorders in Poland (Warsaw, Gdansk) between April 2018 and December 2025. Inclusion criteria were as follows: age ≥ 18 years, confirmed SchS diagnosis according to the validated Strasbourg criteria (5) and treatment with anakinra for at least 2 months. There were no exclusion criteria.

### 2.2 Data sources and extraction

Data were retrieved from electronic medical records and patient charts. The analysis covered demographic, clinical, laboratory and treatment-related data, including sex, month/year of birth, dates of first symptoms and diagnosis, disease manifestations, comorbidities, laboratory investigations (complete blood count - CBC - components, inflammatory markers and monoclonal protein), previous and concomitant therapies, anakinra dosage, treatment duration and any adverse events.

Clinical and laboratory assessments were obtained from routine evaluations at baseline (pre-treatment) and follow-up visits (at 2, 6, 12, 18, 24, 36, 48, 60 and 72 months), when available.

### 2.3 CBC-derived systemic inflammatory indices

Four composite indices were calculated from CBC values (16).

Neutrophil-to-Lymphocyte Ratio (NLR) = neutrophil count/lymphocyte count.

Systemic Immune-Inflammation Index (SII) = (platelet count × neutrophil count)/lymphocyte count.

Systemic Inflammation Response Index (SIRI) = (neutrophil count × monocyte count)/lymphocyte count.

Aggregate Index of Systemic Inflammation (AISI) = (platelet count × neutrophil count × monocyte count)/lymphocyte count.

### 2.4 Ethics

This study was conducted in accordance with the Declaration of Helsinki and approved by the Bioethics Committee of the Military Institute of Medicine (approval no. 51/WIM/24). All patients provided informed consent for the use of their anonymized data for research purposes. Blood sampling and treatment were components of routine clinical practice.

### 2.5 Statistical analysis

Continuous variables are presented as median with interquartile range (IQR; 25th and 75th percentiles) and categorical variables as numbers with percentages. Given the rare−disease context and the observed non−Gaussian, potentially skewed distributions, we chose the median and IQR as robust summaries of central tendency and dispersion, less sensitive to outliers and small samples. Early treatment response was assessed using paired Wilcoxon signed-rank tests. Longitudinal changes over time were evaluated with Friedman’s test for repeated measures with *post-hoc* paired comparisons where appropriate. For biomarker discrimination between active disease and remission, receiver operating characteristic (ROC) curves were generated, areas under the curve (AUCs) calculated with 95% confidence intervals and compared using DeLong’s test. Optimal thresholds were identified using the Youden index with a sensitivity analysis employing patient-level cross-validation. Correlations between biomarkers were evaluated with Spearman’s rank correlation coefficient. Correlation matrices were visualized as heatmaps. Multiple testing was controlled using Holm-Bonferroni adjustments. Data analysis was performed using GraphPad Prism software (version 10.6.1; GraphPad, San Diego, CA, USA) and Stata software (version 18; StataCorp, College Station, TX, USA). All tests were two-sided and a p value < 0.05 was considered statistically significant.

## 3 Results

### 3.1 General characteristics

A total of 13 patients diagnosed with SchS were identified from the medical records of tertiary reference centers for autoinflammatory disorders in Poland. Their baseline demographic and clinical characteristics are summarized in **Table 1**. The cohort consisted of 7 (54%) men and 6 (46%) women (a male-to-female ratio of about 1.2:1). The mean age at disease onset for these patients was 55.8 ± 10.2 years (range: 34–70).

**Table 1:** Patients characteristics.

Variable	Value
Sex, n (%).	
Men	7 (54%)
Women	6 (46%)
Disease characteristics	
Age at disease onset (years) [median, IQR]	58 [48-64]
Baseline features, n (%)	
Urticarial lesions	13 (100%)
Fever	10 (77%)
Fatigue	11 (85%)
Joint/bone pain	12 (92%)
Weight loss	4 (31%)
Lymphadenopathy	2 (15%)
Anemia	6 (46%)
Baseline BMI (kg/m^2^) [median, IQR]	26.4 [25.4–28.3]
Normal	2 (15%)
Overweight	10 (77%)
Obesity	1 (8%)
Baseline laboratory, [median, IQR]	
CRP, mg/dl (ref. < 0.5)	6.9 [2.7-7.1]
SAA, mg/dl (ref. < 0.64)	40.60 [22.30-52.20]
ESR, mm/hr (ref. 0-8)	61.0 [21.0-80.0]
Leucocytes, G/L (ref. 4.30-9.64)	11.02 [7.93-14.57]
Hemoglobin, g/dl (ref. 13.5-17.0)	12.1 [11.7-14.0]
Platelet, G/L (ref. 163-347)	355.0 [245.0-484.0]
Ferritin, ng/mL (ref. 30-400)	192.0 [162.0-217.0]
Procalcitonin, ng/mL (ref. <0.046)	0.09 [0.04-0.24]
Fibrinogen, mg/dl (ref. 200-400)	439.0 [396.0-657.5]
D-dimers, µg/ml (ref. 0-0.5)	0.46 [0.27-0.81]
Monoclonal component, n (%)	
IgM (overall)	12 (92%)
IgM kappa	8 (62%)
IgM lambda	3 (23%)
IgM kappa + lambda	1 (8%)
IgG	1 (8%)
IgG kappa	1 (8%)

BMI, body mass index; CRP, C-reactive protein; ESR, erythrocyte sedimentation rate; IgG, immunoglobulin G; IgM, immunoglobulin M; IQR, interquartile range; n, number; ref., reference range; SAA, serum amyloid A.

### 3.2 Symptoms

Consistent with the diagnostic criteria for SchS, all patients exhibited urticarial exanthema. These lesions were recurrent, non-pruritic and erythoedematous, persisting for more than 24 hours and resolving without bruising or residual hyperpigmentation. Additionally, one patient experienced episodes of angioedema associated with the urticarial rash. The most common systemic manifestations in our cohort included bone pain (12; 92%), fatigue (11; 85%) and fever (10; 77%). Anemia was detected in 6 (46%) patients, although only one patient had a hemoglobin level below 10 g/dL. Other notable findings included weight loss (4; 31%) and lymphadenopathy (2; 15%).

### 3.3 Laboratory

At baseline, all patients presented elevated Erythrocyte Sedimentation Rate (ESR) as well as increased levels of C-reactive protein (CRP) and serum amyloid A (SAA). The median serum CRP concentration was 6.9 [2.7-7.1] mg/dL (reference laboratory value < 0.5 mg/dL), the median SAA 40.60 [22.30-52.20] mg/dL (reference laboratory value < 0.64 mg/dL) and ESR was 61.0 [21.0-80.0] mm/hr. Nine (69%) patients had leucocytosis and 5 (38%) increased platelet counts. Other inflammatory markers were also elevated: fibrinogen in 8 (62%) patients, procalcitonin in 7 (54%), D-dimers in 5 (38%) while ferritin was increased in 2 patients (15%). Monoclonal gammopathy was present in all patients, with IgM being the predominant isotype detected in 12 out of 13 patients (92%). One patient (8%) presented with an IgG kappa monoclonal gammopathy.

### 3.4 Diagnostic delay

All patients fulfilled the Strasbourg diagnostic criteria for SchS. The delay from first symptom onset to SchS diagnosis remained prolonged, with a median of 49.0 months [28.0–56.0]. However, it showed mild improvement in recent years. Mean delay decreased from 54 months (for diagnoses made before 2019) to 36 months (in 2022–2025; the period of the COVID-19 pandemic has been excluded from this analysis), p < 0.05. Prior to referral to a clinical immunologist, patients were most seen by dermatologists (11; 85%), hematologists (10; 77%) and rheumatologist (10; 77%), with much less frequent consultations with allergologists (3; 25%) and nephrologists (1; 8%).

### 3.5 Treatment prior to anakinra

Prior to the initiation of anti-IL-1 targeted therapy, patients were treated with NSAIDs (8; 62%), corticosteroids (12; 92%), antiallergic drugs (11; 85%), immunomodulatory agents (3; 23%) and conventional immunosuppressants (4; 31%). One patient (8%), whose clinical course was previously reported (17), had prior exposure to biologic therapy (tocilizumab). Details for each treatment category are presented in **Table 2**.

**Table 2:** Summary of pharmacological treatments.

Treatment	N (%)
NSAIDs	8 (62%)
Corticosteroids	12 (92%)
Antiallergic	
Antihistamines	11 (85%)
Montelukast	2 (15%)
Immunomodulatory agents	
Colchicine	1 (8%)
Dapsone	3 (23%)
Immunosuppressants	
Azathioprine	1 (8%)
Ciclosporin	1 (8%)
Methotrexate	3 (23%)
Biologics	
Anakinra	13 (100%)
Tocilizumab	1 (8%)

NSAIDs, nonsteroidal anti-inflammatory drugs.

### 3.6 Treatment with anakinra

The average time from disease onset to anakinra initiation was 50.8 [29.1-62.0] months, whereas it was only 1.9 [1.2-4.0] months from the time of SchS diagnosis. All patients presented with active disease upon starting anakinra and showed substantial improvement in clinical signs of disease activity (skin changes, fever, fatigue, musculoskeletal symptoms) within 48 hours. Resolution of symptoms was accompanied by normalization of plasma inflammatory parameters. After 8 weeks of treatment (first laboratory control), the median CRP concentration decreased from 6.9 [2.7-7.1] to 0.1 [0.1-0.4] mg/dL (p < 0.01), SAA from 40.60 [22.30-52.20] to 0.50 [0.40-0.70] mg/dL (p < 0.01), ESR from 61.0 [21.0-80.0] to 23.0 [11.0-33.0] mm/hr (p < 0.01) and the mean hemoglobin level increased from 12.1 [11.7-14.0] to 14.4 [12.8-14.6] g/dL (p < 0.01). All patients achieved complete clinical and laboratory remission. Among five patients (38%) receiving corticosteroids at anakinra treatment initiation, all successfully discontinued steroids during therapy.

**Figure 1:**
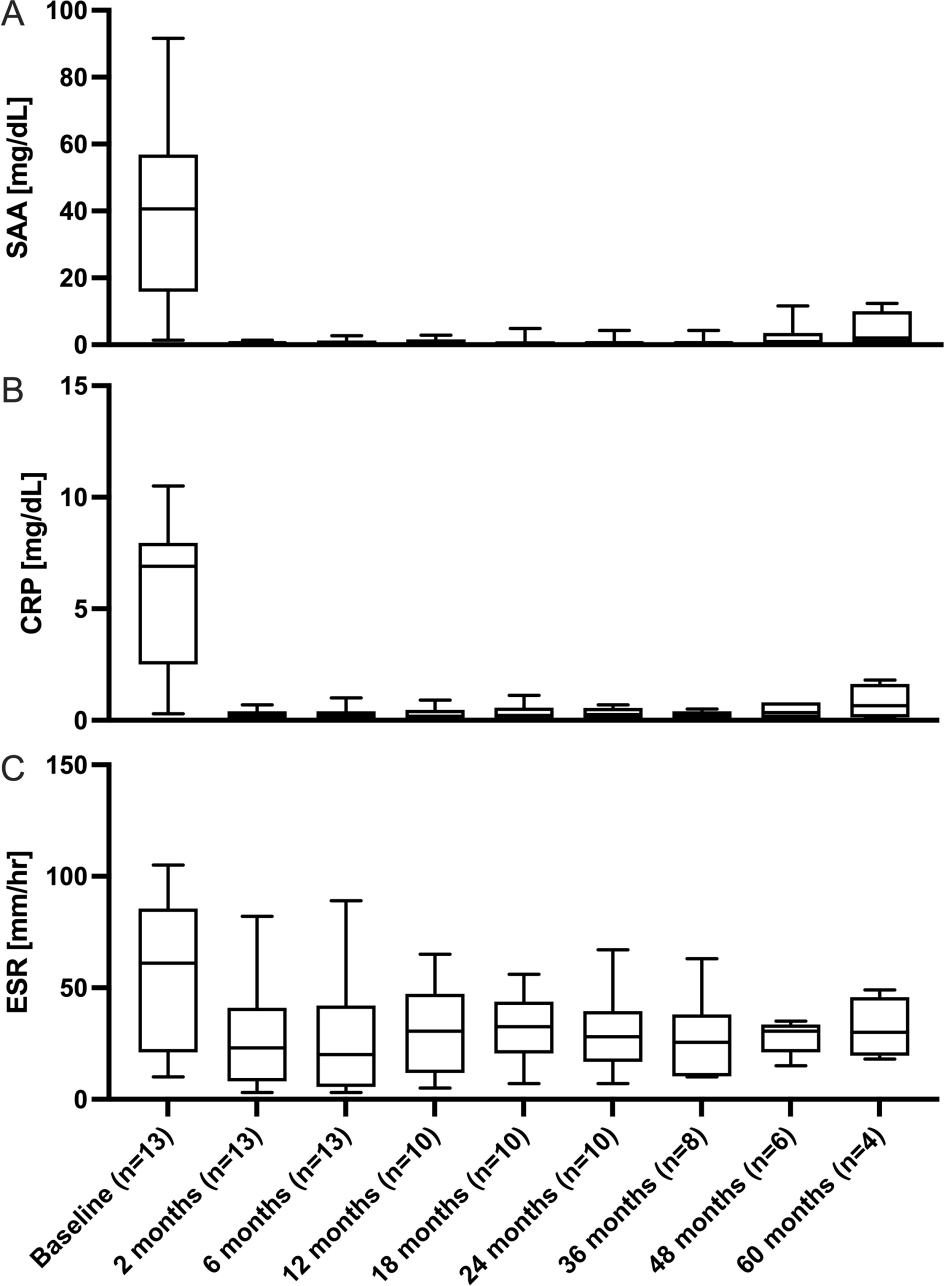
Longitudinal changes in inflammatory parameters during anakinra therapy. **(A)** Serum amyloid A (SAA, mg/dL), **(B)** C−reactive protein (CRP, mg/dL), and **(C)** erythrocyte sedimentation rate (ESR, mm/hr) measured before treatment (Baseline) and at 2, 6, 12, 18, 24, 36, 48, and 60 months of follow−up. Each box represents the median (horizontal line) and interquartile range (IQR; 25th–75th percentiles). Whiskers indicate the minimum and maximum observed values at a given time point. The number of evaluable patients **(n)** for each time point is shown on the x−axis.

Ten patients (77%) completed ≥ 24 months of follow-up, 8 (62%) ≥ 36 months with a median overall follow-up of 38.2 months [32.9–71.7] and a maximum of 92.5 months. During this follow-up the efficacy of anakinra remained unchanged. The long-term stability of inflammatory parameters over time is illustrated in **Figure 1**. None of the patients discontinued anakinra during observation.

All patients were started on an initial dose of 100 mg daily, without requirement for dose escalation. In 3 patients (23%), administration frequency was successfully reduced by extending the injection interval to every 2 days (2 patients) or 3 days (1 patient).

Regarding disease complications, anakinra had no impact on the monoclonal component level and no patients progressed to a hematologic malignancy. One patient, who had been diagnosed with AA amyloidosis and chronic renal disease before starting anakinra, demonstrated stabilization of her condition without further progression of renal dysfunction during therapy.

### 3.7 Safety of anakinra

Treatment with anakinra was generally well tolerated, with no serious adverse events reported in our cohort. Injection site reactions occurred in 7 of 13 patients (54%) during the first weeks of treatment and resolved spontaneously. No anaphylactic reactions were observed. Two patients (15%) developed mild paradoxical psoriasis that improved with topical corticosteroids. One patient (8%) experienced transient neutropenia. Minor infections, primarily respiratory or urinary tract infections, occurred during the therapy. All adverse events resolved with standard treatment and did not require discontinuation of anakinra.

Notably, all patients experienced weight gain with a median increase of +6.0 kg [3.0–9.0] (+6.82% [5.19–11.67]; p<0.01), independent of age, sex or baseline weight. Median BMI increased from 26.40 [25.36–28.28] to 28.62 [27.06–29.89] with obesity prevalence rising from 7.7% (n=1) to 23.1% (n=3). Other participants were classified as normal weight - 2 patients (15.4%) while 8 as overweight (61.5%).

### 3.8 Biomarkers of disease activity

**Figure 2:**
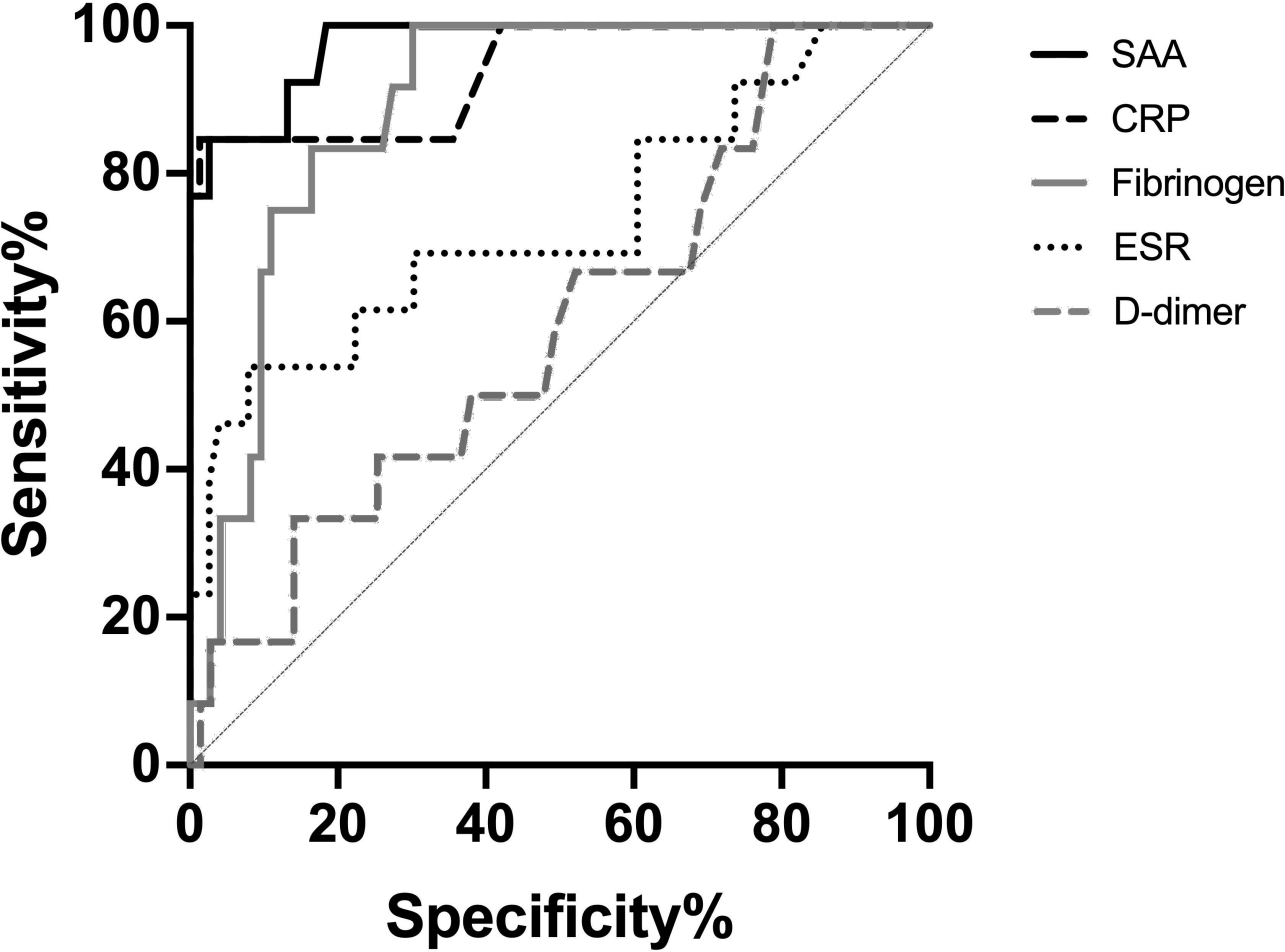
Receiver operating characteristic (ROC) curves summarize the trade−off between sensitivity and specificity across decision thresholds for the biomarkers SAA, CRP, fibrinogen, ESR (erythrocyte sedimentation rate) and D−dimer in assessing disease activity. The upper−left proximity of a curve reflects greater discriminative ability; the diagonal reference line represents a non−discriminatory classifier (AUC = 0.5).

In ROC analyses comparing active disease with clinical remission, SAA demonstrated the highest discriminatory power (AUC 0.974, 95% CI 0.940–1.000), followed by CRP (AUC 0.940, 95% CI 0.860–1.000) and fibrinogen (AUC 0.890, 95% CI 0.816–0.963); ESR showed moderate performance (AUC 0.733, 95% CI 0.562–0.904), while D-dimer performed poorly (AUC 0.593, 95% CI 0.422–0.765) - **Figure 2**. Using the Youden index, an SAA threshold of ≥1.4 mg/dL provided 100% sensitivity and 82% specificity for identifying baseline activity, whereas CRP ≥2.30 mg/dL achieved 84.6% sensitivity and 100% specificity. There was no statistically significant difference between SAA and CRP in AUC comparison (DeLong p = 0.20). SAA outperformed fibrinogen (p=0.043), ESR (p<0.001) and D-dimers (p<0.001). In patient-level cross-validation, SAA remained the strongest single biomarker (AUC 0.933). CRP alone showed lower discriminatory power (AUC 0.867), although this difference was not statistically significant (p = 0.32).

### 3.9 CBC-derived systemic inflammatory indices

**Figure 3:**
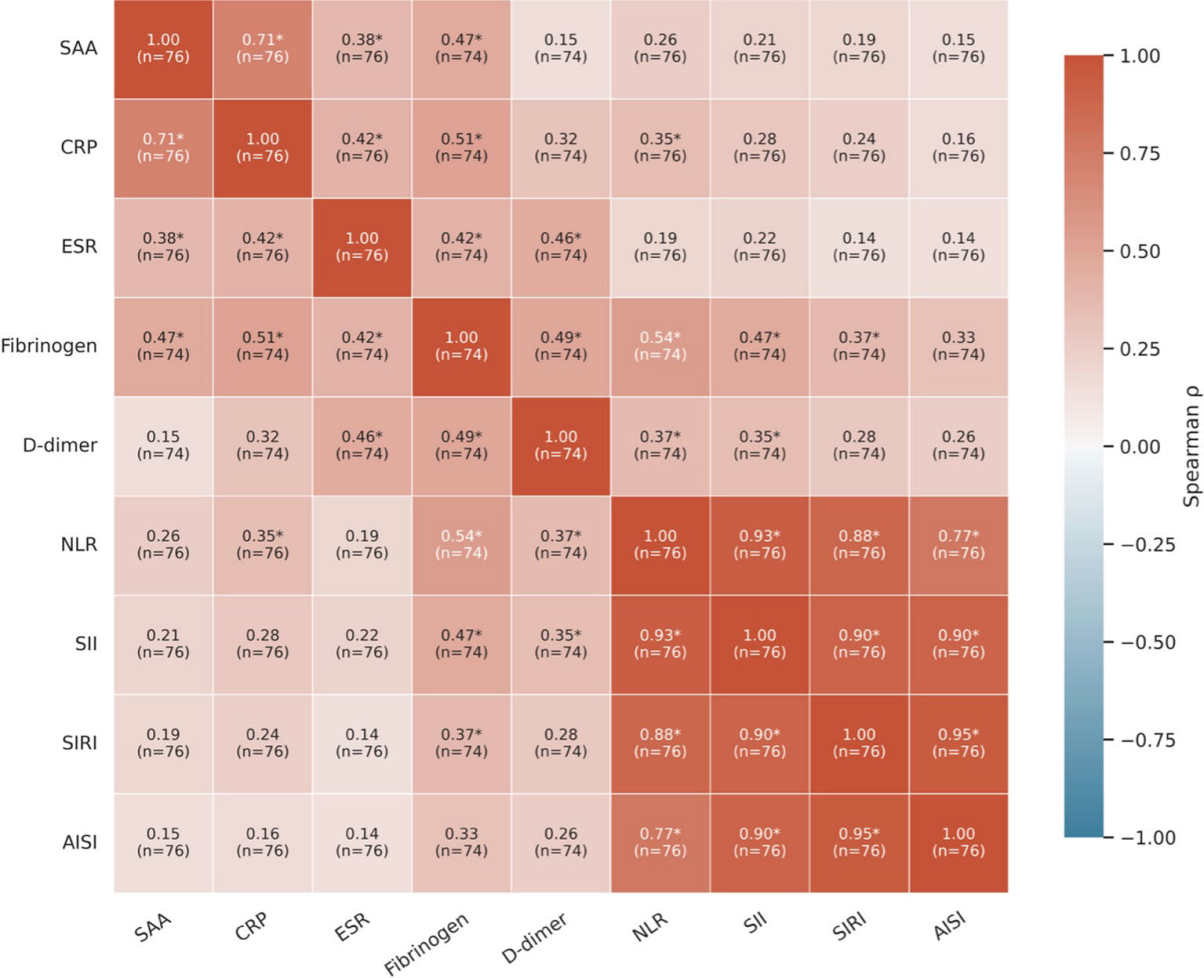
Heatmap of pooled Spearman correlations across all timepoints for evaluated inflammatory parameters. The matrix displays Spearman’s rank correlation coefficients (ρ) between classical acute-phase reactants (SAA, CRP, ESR, Fibrinogen, D-dimer) and complete blood count (CBC)-derived inflammatory indices (NLR, SII, SIRI, AISI). Each cell contains the correlation coefficient and the corresponding sample size (n). The color gradient reflects the strength and direction of the correlation, with darker shades of red indicating stronger positive correlations. Asterisks (*) denote statistical significance (p < 0.05) after adjusting for multiple comparisons.

All four composite hematologic indices NLR, SII, SIRI and AISI showed a marked decline after anakinra treatment initiation, with subsequent stabilization during long-term follow-up (**Table 3**). Friedman’s test limited to time points with complete longitudinal (baseline, 2, 6, 12, 18, and 24 months; n = 10), confirmed a significant time effect for all indices (p < 0.05). The most pronounced reduction occurred within the first 2 months, indicating of broad suppression of systemic inflammation. Specifically, median NLR decreased from 3.52 [1.90–5.35] to 1.36 [1.06–2.02], SII from 1791.85 [464.59–2086.97] to 491.86 [206.27–709.03], SIRI from 1.99 [1.46–4.98] to 1.13 [0.53–1.50] and AISI from 682.15 [337.67–1988.94] to 329.54 [99.01–517.74]. At baseline (corresponding to clinically active disease), SAA correlated significantly with NLR (ρ = 0.61, p = 0.0269) and SII (ρ = 0.58, p = 0.0367), while SIRI and AISI showed weaker, non-significant associations (SIRI: ρ = 0.49, p = 0.0899, AISI: ρ = 0.48, p = 0.0941). To assess whether early changes in these inflammatory indices tracked the biochemical response, correlations between ΔSAA and Δindices (baseline to 2 months) were examined. Absolute changes in SII demonstrated moderate correlations with SAA reductions (ρ = 0.55, p = 0.049), suggesting that this index track treatment response, however less dynamically than SAA. Associations for NLR (ρ = 0.50, p = 0.082), SIRI (ρ = 0.36, p = 0.223) and AISI (ρ = 0.51, p = 0.074) did not reach statistical significance. A pooled correlation heatmap including conventional inflammatory biomarkers and the four CBC-derived inflammatory indices demonstrated a coherent inflammatory network (**Figure 3**). SAA showed strong positive correlations with CRP and moderate correlations with ESR and fibrinogen. No statistically significant correlation was observed between the CBC-derived inflammatory markers (NLR, SII, SIRI, AISI) and SAA after adjustment for multiple comparisons.

**Table 3:** Values of CBC-derived inflammatory indices at each timepoint, with number of patients (n).

Time point (number of patients)	NLR	SII	SIRI	AISI
Before treatment (n=13)	3.52 [1.90–5.35]	1791.85 [464.59–2086.97]	1.99 [1.46–4.98]	682.15 [337.67–1988.94]
2 months (n=13)	1.36 [1.06–2.02]	491.86 [206.27–709.03]	1.13 [0.53–1.50]	329.54 [99.01–517.74]
6 months (n=13)	1.40 [1.09–1.88]	368.25 [297.08–542.35]	0.81 [0.71–0.95]	217.27 [146.05–269.39]
12 months (n=10)	1.33 [0.95–1.73]	350.26 [265.99–438.85]	0.92 [0.66–1.11]	234.05 [170.72–293.36]
18 months (n=10)	1.58 [0.98–2.31]	396.40 [260.51–599.85]	0.79 [0.55–1.46]	175.27 [150.91–482.31]
24 months (n=10)	1.33 [0.90–1.68]	359.58 [210.06–478.56]	0.89 [0.42–1.09]	206.64 [95.47–301.49]
36 months (n=8)	1.37 [1.15–1.53]	351.34 [308.65–405.89]	0.79 [0.67–0.98]	195.97 [161.41–241.25]
48 months (n=6)	1.17 [0.74–1.42]	287.34 [193.59–383.42]	0.63 [0.56–1.16]	153.08 [149.16–225.60]
60 months (n=4)	1.25 [0.96–1.47]	274.81 [234.48–329.54]	0.95 [0.84–0.99]	222.16 [178.38–262.81]
72 months (n=2)	2.12 [1.79–2.44]	641.88 [555.16–728.60]	1.61 [1.39–1.83]	489.04 [431.89–546.19]

Values are presented as median [interquartile range]. CBC, complete blood count; NLR, neutrophil-to-lymphocyte ratio; SII, systemic immune-inflammation index; SIRI, systemic inflammation response index; AISI, aggregate index of systemic inflammation.

## 4 Discussion

This study provides a comprehensive real-world analysis of anakinra treatment in a cohort of Polish patients with SchS, providing unique insights into its long-term efficacy, safety profile and the utility of various biomarkers for monitoring this rare autoinflammatory disease. Our findings reinforce the key role of IL-1 blockade, highlighting important clinical challenges requiring careful consideration, especially dosing optimization and the nuanced interpretation of inflammatory markers.

All 13 patients in our cohort achieved complete clinical remission within 48 hours of initiating anakinra treatment, characterized by the resolution of fever, skin lesions, musculoskeletal pain and fatigue. This prompt response can serve as a diagnostic confirmation and aligns with observations in other autoinflammatory syndromes, where IL-1β functions as a central pathogenic mediator (18, 19). In presented cohort, the clinical and biochemical responses to anakinra were sustained over a median follow-up of 38.2 months (longest 92.5 months), confirming its long-term efficacy in controlling SchS activity. This extended observational period with structured follow-up assessments and extending beyond 7 years in some patients, provides valuable real-world evidence on the therapeutic advantages of anakinra, extending current literature. Sustained symptom control and persistent normalization of inflammatory markers support the feasibility of a treat-to-target remission strategy in SchS. The corticosteroid-sparing effect was also clinically relevant. All patients receiving glucocorticoids successfully tapered and discontinued steroids during maintenance IL-1 blockade. Our findings are consistent with a French multicenter observational series, in which all 29 anakinra treated SchS patients showed rapid improvement, typically within 48 hours. Treatment effectiveness remained unchanged over a median of 36 months (range 3–79), with 73.1% patients achieving complete and 29.9% partial remission (20). Similarly, a UK series reported resolution of all symptoms with CRP normalization in 19 out of 20 (95%) patients, sustained over a median treatment duration of 60 months (range, 15–115 months) without discontinuations (21). The efficacy of anakinra has been broadly documented in the international AIDA Network registry, which includes also patients from our center (13). The rapid clinical remission observed in these patients underscores the value of anakinra not just as a targeted treatment, but also as a practical diagnostic test in patients with suspected SchS.

Optimal dosing strategies for anakinra in SchS remain a subject of clinical interest. None of our patients required dose escalation above the standard 100 mg daily, in contrast to some earlier reports suggesting that higher doses, switch to canakinumab or tocilizumab may be necessary in some patients to achieve complete clinical and biochemical remission (22–24). A key finding from our study is the possibility of dose reduction in carefully selected patients. In our cohort, 3 patients in sustained remission successfully extended injection intervals to every 48–72 hours without loss of disease control. This observation has important practical implications, as reducing injection frequency may improve long-term acceptance and adherence while potentially decreasing treatment burden and associated costs. Although similar interval extension approaches have been reported in other autoinflammatory conditions treated with IL-1 blockade (25, 26), evidence specific to SchS remains limited. Existing data indicate that interval extension is not universally successful and should be approached as an individualized trial rather than a routine strategy (20, 27). This approach also carries a risk of disease flare, as evidenced by the French multicenter experience, where only 4 out of 19 patients maintained complete clinical remission on alternate-day dosing, while the remainder had to resume daily injections (20). Based on our experience dose reduction (by extending the dosing interval) may be considered only in patients with sustained clinical remission and stable normalization of inflammatory markers. A practical information before planned tapering is assessment of tolerance to an unplanned missed dose, which may help anticipate the likelihood of successful de-intensification. If pursued, interval extension should be implemented gradually, with close monitoring during the initial weeks for disease flares and prompt dose re-escalation if needed.

The safety profile of anakinra in our cohort was favorable, with no serious adverse events reported. This reinforces its well-established safety records and aligns with previous studies demonstrating good tolerability during long-term use (13, 20, 21, 25, 28). Injection site reactions were the most frequent adverse events, observed in 54% of patients. However, these were generally mild, transient and did not lead to treatment discontinuation - consistent with experiences from other SchS cohorts and product-label data (20, 29). Minor respiratory and urinary tract infections were also recorded, but all resolved with standard treatment and did not require discontinuation of anakinra. These findings should be interpreted in the context of routine safety monitoring during anakinra therapy, including vigilance for infections, periodic blood count assessment because of the risk of neutropenia and attention to renal and hepatic function (30). Overall, our results further support the favorable tolerability profile of anakinra in patients with SchS.

Two cases of paradoxical psoriasis were observed in our cohort, representing a rare but significant adverse event that warrants further investigation. Anakinra-induced psoriasis has been reported previously, even in patients without a prior history of psoriatic disease (31–33). Although paradoxical psoriasiform form eruptions are well described with other biologic therapies, especially TNF inhibitors, the pathways driving such reactions after IL-1 blockade are still not fully defined (34, 35). Inhibition of IL-1 signaling may unmask or promote alternative inflammatory cascades, including TNF-type I interferon and IL-23/IL-17 axes, which are central to psoriasis pathogenesis (36, 37). It was shown that lesions in plaque psoriasis exhibit an overexpression of endogenous IL-1ra, with an 10-fold increase in the IL-1ra to IL-1α ratio compared with normal skin (38, 39). Further studies are needed to clarify the immunological basis of these paradoxical events and to identify patients at risk.

A noteworthy clinical observation in our cohort was the consistent weight gain observed across patients. This median increase of 6 kg led to a concerning shift in BMI distribution. Importantly, weight gain occurred independently of age, sex or baseline weight, suggesting a systemic effect of IL-1 blockade. The most likely mechanism underlying this observation is the resolution of the chronic catabolic state driven by uncontrolled autoinflammation. In chronic systemic inflammation, IL-1 acts centrally on the hypothalamus to suppress appetite and peripherally to increase resting metabolic rate (40). Patients with SchS frequently experience weight loss during active or untreated disease phases, as reported in 60% of the French multicenter cohort and 47% of the UK series (20, 21). Once inflammation is controlled by IL-1 suppression, appetite may increase and the hypermetabolic state resolves. If caloric intake remains unchanged, this metabolic shift favors a positive energy balance, leading to gradual weight gain over subsequent months. Similar trends have been documented in other IL-1 driven conditions, including cryopyrin-associated periodic syndromes and familial Mediterranean fever, where effective disease control can paradoxically lead to weight gain (41, 42). Furthermore, IL-1 plays a complex role in glucose and lipid metabolism. While chronic low-grade inflammation is typically associated with insulin resistance, the rapid suppression of the hyperinflammatory state may alter adipocyte function, attenuating thermogenic and lipolytic signals, thereby promoting lipid storage (43, 44). Clinicians should discuss potential weight gain upon anakinra initiation, emphasizing the importance of dietary awareness and regular physical activity. Baseline weight and BMI should be documented with periodic monitoring during follow-up to identify significant trends early. The potential impact of BMI on the clinical efficacy of anakinra is a relevant consideration in the management of autoinflammatory disorders. Pharmacokinetic studies in healthy volunteers found that in heavier individuals, the volume of distribution and systemic clearance of anakinra can increase, which theoretically might lower overall drug exposure (45). However, because the absolute bioavailability of subcutaneous anakinra remains consistently high and the drug possesses a wide therapeutic window, these pharmacokinetic changes rarely translate into a loss of clinical efficacy. In our cohort, all patients achieved a full response to standard anakinra dosing regardless of their BMI, and no dose escalations were required for overweight or obese individuals. Consistent with product labeling, adult dosing is fixed (weight-based dosing applies to pediatric/CAPS indications) and routine BMI-based adjustment is not recommended.

One of the findings of our study is the superiority of SAA over other inflammatory markers in differentiating active disease from remission. While CRP also demonstrated strong performance, SAA emerged as the most robust single biomarker based on patient-level cross-validation. CRP and SAA are generally strongly correlated across broader autoinflammatory disease cohorts, but clinically meaningful discordance can occur (46, 47). CRP’s universal availability, cost-effectiveness and reliance on highly standardized assays make it pragmatic for general disease tracking, yet it may lack sensitivity for low-grade inflammation. Conversely, recent expert consensus and cohort studies in autoinflammatory diseases state that SAA, despite lesser assay standardization, is sensitive and specific indicator of residual or subclinical inflammation and is directly correlated with long-term AA amyloidosis risk (9, 47–50). The physiological rationale for SAA superiority in SchS stems from their distinct regulation by cytokines. Specifically, SAA production is more strongly stimulated by IL-1, whereas CRP is mainly driven by IL-6 (51, 52). Furthermore, SAA has a shorter half-life than CRP (approximately 34.9 ± 28.7 hours vs 46.4 ± 21.7 hours), allowing for more rapid detection of changes in inflammatory activity (53). This property is particularly important in patients experiencing partial symptom recurrence, those undergoing dose-interval modifications or when objective confirmation of remission is required.

Our study is among the first to systematically analyze CBC-derived inflammatory indices (NLR, SII, SIRI, AISI) in the context of SchS. All four indices showed significant declines after anakinra initiation with the most pronounced reduction occurring within the first 2 months. In active disease, SAA correlated moderately with NLR and SII, suggesting that these simple indices reflect the systemic inflammatory burden in SchS. Nevertheless, after correction for multiple comparisons, these associations did not remain statistically significant as well as the dynamic changes over time (ΔSAA vs. Δindices) were weak. This discrepancy can largely be attributed to the distinct kinetic profiles of these markers. While SAA is directly and rapidly induced by IL-1, with a half-life of hours, CBC-derived indices depend on bone marrow dynamics and cellular turnover (53, 54). Neutrophils and platelets equilibrate much more slowly in the circulation (54). Consequently, changes in CBC parameters lag behind the rapid fluctuations of SAA during both disease flares and immediate responses to IL-1 blockade. In recent years, CBC-derived indices have gained attention as simple, economical tools for assessing inflammatory status across various disorders, ranging from malignancies to autoimmune diseases (16, 55). Our data suggest a more limited role in SchS. These indices do not replace direct acute-phase markers such as SAA or CRP. Instead, CBC-derived indices may serve as auxiliary markers of general inflammation, making them useful in situations where specific inflammatory markers are not routinely available. Their strengths include accessibility, cost-effectiveness and immediate availability in routine care, whereas their main disadvantage involve a slower dynamic response, susceptibility to confounding factors and lack of specificity for IL-1-driven autoinflammation (16, 55, 56).

Several limitations due to the retrospective design of this study should be acknowledged. First, reliance on routine clinical visits and varying treatment initiation times resulted in uneven follow-up durations across patients (only two reached the follow up after 72-month). Second, the absence of standardized patient-reported outcome measures precluded formal quantification of the treatment’s impact on quality of life, despite all patients reporting subjective clinical improvement. Third, detailed data on the duration of individual therapies used before anakinra initiation were not consistently available in the registry, which limited a more precise analysis of treatment exposure before IL-1 blockade. Nevertheless, the types of prior treatments are summarized in **Table 2** and treatment delay is additionally reflected by the time from first disease symptoms to anakinra initiation and by the time from SchS diagnosis to anakinra initiation. Finally, while the sample size of 13 patients limits the statistical power for complex subgroup analyses, it is crucial to highlight that for an ultra-rare condition like Schnitzler syndrome, this represents one of the largest cohorts described in the literature to date. Despite these limitations, our findings translate into several important implications for clinical practice.

## 5 Conclusions

This Polish real-world cohort demonstrates that anakinra provides rapid and sustained clinical remission of Schnitzler syndrome, with consistent suppression of systemic inflammation and a favorable long-term safety profile. These findings strongly support anakinra as first-line therapy initiated immediately upon diagnosis. Among evaluated biomarkers, SAA emerged as the most reliable indicator of active disease, positioning it as the preferred marker for longitudinal monitoring in SchS. CBC-derived inflammatory indices declined substantially post-treatment and offer accessible supplementary information. However, they should be regarded as complementary adjuncts rather than substitutes for acute-phase reactants. A significant observation was the marked diagnostic delay, highlighting an ongoing need for enhanced cross-specialty awareness and earlier recognition of the characteristic clinical phenotype. Furthermore, long-term patient management must account for specific treatment-emergent effects; clinicians should actively monitor and counsel patients regarding the consistent weight gain as well as the potential for paradoxical anakinra-induced psoriasis. Finally, in patients achieving stable remission, cautious extension of injection intervals may be considered in carefully selected individuals under rigorous clinical and laboratory oversight.

## Data Availability

The raw data supporting the conclusions of this article will be made available by the authors, without undue reservation.
